# Determinants of therapy failure among adults on first-line antiretroviral therapy in Asmara, Eritrea: a multicenter retrospective matched case–control study

**DOI:** 10.1186/s12879-022-07797-2

**Published:** 2022-11-10

**Authors:** Samuel Tekle Mengistu, Ghirmay Ghebrekidan Ghebremeskel, Hermon Berhe Ghebrat, Oliver Okoth Achila, Nahom Asmerom Yohannes, Amon Solomon Ghebrenegus, Filmon Ghebretsadik Wendmhuney, Naod Yeibyo, Amanuel Kidane Andegiorgish, Araia Berhane Mesfin, Negassi Leake

**Affiliations:** 1 Nakfa Hospital, Ministry of Health Northern Red Sea Branch, Nakfa, Eritrea; 2 Af’abet Hospital, Ministry of Health Northern Red Sea Branch, Nakfa, Eritrea; 3 Unit of Clinical Laboratory Science, Orotta College of Medicine and Health Sciences, Asmara, Eritrea; 4 Gedem Naval Hospital, Gedem, Eritrea; 5 Hazhaz Zonal Referral Hospital, Asmara, Eritrea; 6 Halibet National Referral Hospital, Asmara, Eritrea; 7 Ghindae Zonal Referral Hospital, Ministry of Health Northern Red Sea Branch, Ghindae, Eritrea; 8 Department of Epidemiology and Biostatistics, School of Public Health, Xi’an Jiaotong University Health Science Center, Xi’an, People’s Republic of China; 9 Communicable Disease Control Division, Ministry of Health, Asmara, Eritrea; 10 Department of Internal Medicine, Orotta College of Medicine and Health Sciences, Asmara, Eritrea

**Keywords:** CD4^+^ cell count, HIV, Treatment failure, Viral load, Eritrea

## Abstract

**Background:**

Information on treatment failure (TF) in People living with HIV in a data-poor setting is necessary to counter the epidemic of TF with first-line combined antiretroviral therapies (cART) in sub-Saharan Africa (SSA). In this study, we examined the risk factors associated with TF in Asmara, Eritrea from 2001 to 2020.

**Methods:**

A multicenter, retrospective 1:2 matched (by age and gender) case–control study was conducted in four major hospitals in Asmara, Eritrea on adults aged ≥ 18 years who were on treatment for at least 6 months. Cases were patients who fulfills at least one of the WHO therapy failure criterion during the study period. Controls were randomly selected patients on first-line treatment and plasma viral load < 1000 copies/ml in their latest follow-up measurement. Multivariable logistic regression analysis was conducted to identify risk factors for TF. All *P*-values were 2-sided and the level of significance was set at P < 0.05 for all analyses.

**Results:**

Of the 1068 participants (356 cases; 712 controls), 585 (54.7%) were females. The median age at treatment initiation was 46 years [interquartile range (IQR): 39–51]. Median time to combined antiretroviral therapy (cART) failure was 37 months (IQR = 24–47). In the multivariate analysis, factors associated with increased likelihood of TF included initial nucleoside reverse transcriptase inhibitors (NRTI) backbone (Zidovudine + Lamivudine (AZT + 3TC): adjusted odds ratio (aOR) = 2.70, 95% Confidence interval (CI): 1.65–4.41, P-value < 0.001), (Abacavir + lamivudine (ABC + 3TC): aOR = 4.73, 95%CI: 1.18–18.92, P-value = 0.028], and (Stavudine + Lamivudine (D4T + 3TC): aOR = 5.00; 95% CI: 3.03–8.20, P-value < 0.001) in comparison to Emtricitabine and Tenofovir diproxil fumarate (FTC + TDF). Additional associations included prior exposure to cART (aOR = 2.28, 95%CI: 1.35–3.86; P- value = 0.002), record of sub-optimal drug adherence (aOR = 3.08, 95%CI: 2.22–4.28; P < 0.001), ambulatory/bedridden at presentation (aOR = 1.61, 95%CI: 1.12–4.28; P-value = 0.010), presence of comorbidities (aOR = 2.37; 95%CI: 1.36–4.10, P-value = 0.002), duration of cART (< 5 years: aOR: 5.90; 95% CI: 3.95–8.73, P-value < 0.001), and use of SMX-TMP prophylaxis (aOR = 2.00, 95%CI, 1.44–2.78, P-value < 0.001).

**Conclusion:**

Our findings underscore the importance of optimizing cART adherence, diversification of cART regimens, and interventions directed at enhancing early HIV diagnosis, prompt initiations of treatment, and improved patient-focused monitoring of treatment response.

## Key summary points

Why carry out this study?

- The lack of data regarding the magnitude and possible risk factors for TF in Eritrea makes it difficult to formulate evidence-based improvements in patient management. This study has identified important determinants of therapy failure in Asmara, Eritrea.
- By undertaking an inquiry into the sociodemographic and clinical risk factors associated with TF, we believe that we have gathered useful information that can be used by clinicians to identify patients who are at a higher risk of TF.
- Importantly, conflicting findings have also been reported regarding immunologic recovery after cART initiation, particularly the long-term impact of age (note that patients in this setting are relatively old). Therefore, we examined changes in CD4 recovery between cases and controls.

What was learned from this study?

- The study revealed important clinical risk factors for cART therapy failure.
- The study indicates that treatment response is a multifactorial outcome and control of this epidemic calls for a multifaceted approach involving multiple sectors of the healthcare system in Eritrea. Reduction in late presentation of patients/early diagnosis, prompt treatment initiation, and targeted monitoring and enhancement of adherence.
- In addition, the need for research on determinants of sub-optimal adherence was implied.

## Introduction

In 2020, approximately 37.7 million (95% CI: 30.2–45.1 million) people globally were living with the Human Immunodeficiency Virus (HIV) [1] with adults constituting 36.2 million (95% CI: 30.2–42.5 million). The data also demonstrates that a disproportionate number of people living with HIV (PLWHIV)—25.7 million (~ 69.5%), are in sub-Saharan Africa (SSA). In terms of mortality, approximately 36.3 million [27.2–47.8 million] people have died of HIV/AIDS-related illnesses or complications in the last 3 decades [1], and a disproportionate number of these deaths occurred in SSA. Fortunately, combined antiretroviral therapies (cART), coupled with advances in HIV testing technology have transformed the HIV landscape from a disease with significant long-term morbimortality (2000–2015, ~ 8 million HIV-related deaths were averted) into a relatively manageable chronic condition. For context, the massive global expansion of cART coverage from ~ 7% (7.8 million [6.9 million–7.9 million]) in 2010 to 77% (~ 27.5 million [26.5 million–27.7 million]) in 2020[1] has led to tremendous gains in adult life expectancy. By reducing community viral load, large-scale cART rollout in SSA has also reduced HIV transmission at the population level [2]. Based on these findings, a 2015 update of the World Health Organization (WHO) treatment guideline-recommended treatment for all PLHIV regardless of CD4^+^ cell status [3].

Notwithstanding the observed gains, the downward trend in HIV-associated mortalities appears to have stalled in recent years. Globally, HIV has still associated with approximately 680,000 [480,000–1.0 million] mortalities per year and remains a leading cause of death in specific demographic groups or countries, particularly in SSA [4]. Multiple explanations have been invoked to explain this intractable mortality rate [2]. However, treatment failure (defined either as clinical failure—AIDS-defining event; virologic failure—having two consecutive viral loads > 1000 copies/mL with adherence counseling between measurements (a WHO standard commonly used in Low and medium-income countries (LMIC) in SSA); or immunologic failure—CD4^+^ cell count < 200 mm^3^) is regarded by most experts as the most formidable problem. Others have warned that the rapidly evolving epidemic of cART failure may profoundly undermine existing public health gains [5].

For context, modeling studies predicted that drug resistance mutations (DRMs) to non-nucleoside reverse transcriptase inhibitors (NNRTI) ˃ 10%, could account for 105,000 and 135,000 excess HIV infections and deaths in SSA between 2016 and 2020 [6]. Further, estimates suggest that by 2030, 2 million HIV patients in SSA will be on the costlier second-line cART [1]. Post-2018 changes by the WHO on preferred first-line cART which recommended dolutegravir (DTG) -based regimen may have reduced the likelihood of such outcomes. However, a significant number of patients in SSA are still on previous first-line regimens. This outcome is related to the fact that transition to DTG-based regimens has been hampered by implementation bottlenecks and the fact that the WHO and some national guidelines in SSA still recommend some non-DTG-based regimens as alternative first-line or second-line regimens—Tenofovir (TDF) + Lamivudine (3TC) + Atazanavir/ritonavir (ATV/r) or TDF + 3TC + Efavirenz (EFV) as first or second first-line alternatives [7, 8].

Overall, data regarding the determinants of virologic failure have implicated a combination of factors. These include patient-related factors (sub-optimal adherence to cART—psychosocial factors, co-morbidities, poor linkage to care, interruption of cART, cost, adverse drug reactions/tolerability); viral factors (persistence of drug-resistant strains; secondary resistance/prior failures, higher pre-treatment viral load); and drug-related factors (sub-optimal pharmacokinetics/pharmacodynamics; low genetic barrier (e.g. ARVs which require a limited number of mutations for developments of resistance—3TC, FTC, and EFV, among others), inappropriate cART regimen; Drug-drug interactions with non-ARVs, among others) [9, 10]. In general, varying permutations and combinations of these factors have been uncovered by investigators in SSA [9, 11].

Although access to data on virologic failure from SSA has improved in recent years; information from a a large number of countries is missing. More importantly, program-level differences, such as site-level processes or cultural differences and variations in policies and implementation priorities; limits extrapolation of data to other settings. Eritrea, for instance, is regarded as one of the success stories in SSA when it comes to HIV/AIDS prevention—current estimates suggest that the prevalence of HIV is approximately 0.7% [12]. However, concerns remain regarding patients’ management. Unlike other countries in the region, basic data on treatment failure or other HIV-related themes are unavailable in publicly available repositories. Therefore, the objective of this study was to examine the risk factors associated with cART therapy failure (TF) in Asmara, Eritrea. It’s our opinion that understanding the correlates of first-line treatment failure is essential for patient monitoring and targeted intervention [12, 13].

## Methods

### Study design and setting

**Fig. 1:**
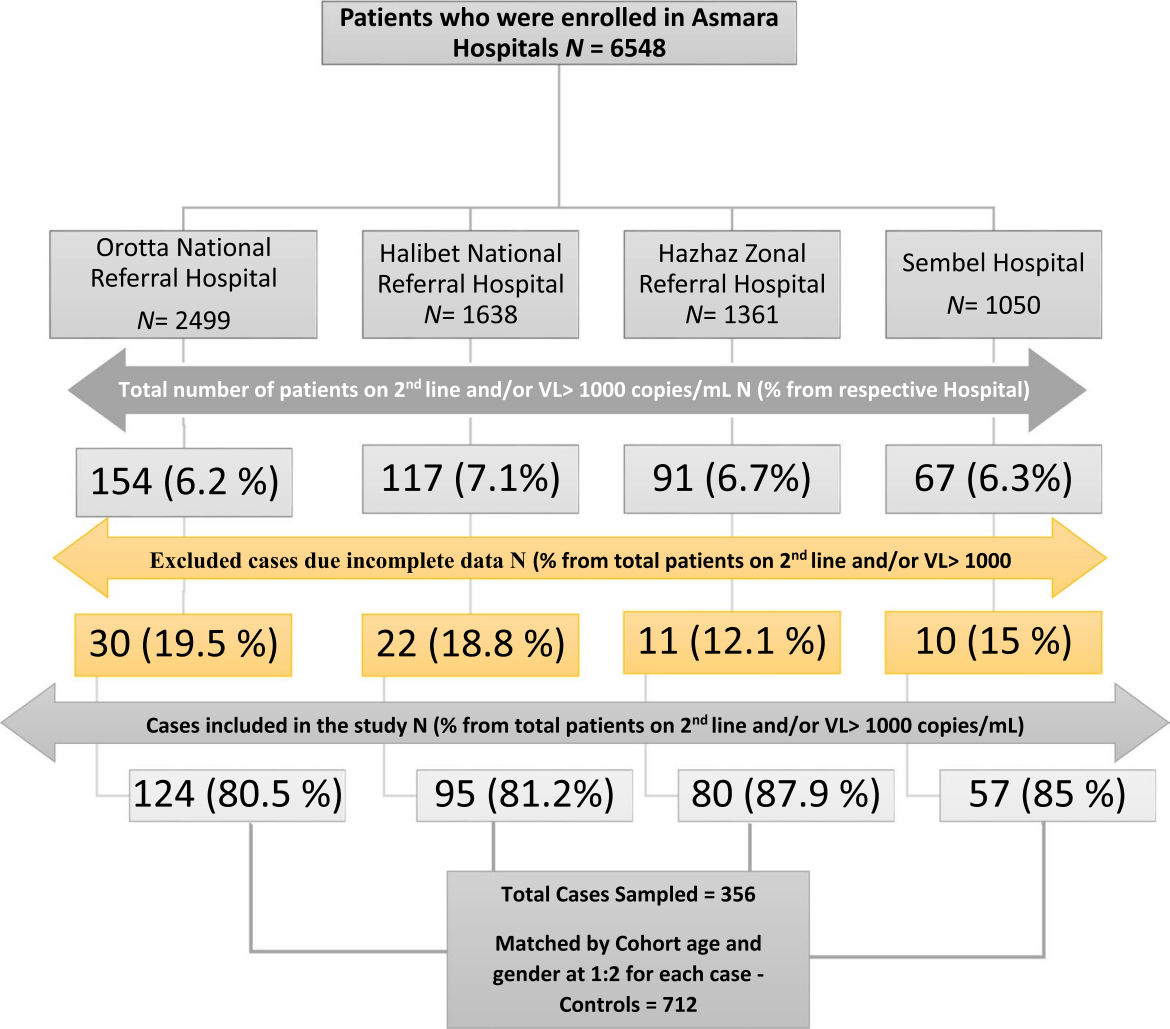
Flow diagram of study participants’ recruitment Outcomes of adults living with HIV in Asmara’s Follow-up hospitals, Asmara, Eritrea (2001–2020)

A retrospective case–control study was carried out to identify the factors associated with virologic failure among adults on first-line antiretroviral therapy. The study was conducted in four major hospitals (Orotta National Referral Hospital (ONRH); Halibet National Referral hospital (HRH); Sembel hospital (SH); and Haz Haz Zonal Referral Hospital (HzH) located in Asmara, the capital city of Eritrea. A total of 6548 patients (2499 in ONRH, 1638 in HRH, 1361 in HzH, and 1050 in SH) have accessed treatment since 2001. See Fig. 1. The four hospitals were the main treatment centers for HIV before the decentralization of HIV treatment services in the country.

### Study population

Adult patients (≥ 18 years of age) who received first-line cART treatment for a period of more than 6 months at one of the four major hospitals in the study period (2001–2020) were eligible for recruitment. Participants with incomplete records were excluded from the analysis. Cases were patients who fulfills at least one of the WHO therapy failure criterion during the study period. Controls were those who doesn’t fulfill any of the WHO therapy failure criterion during the study period. For each case, two matched randomly selected controls were enrolled based on the plasma viral load.

### Sample size determination and sampling procedure

We extracted all records from the registry from the year 2001 to 2020. A total of 356 patients from the four hospitals (ONRH = 124, HH = 95, HzH = 80, and SH = 57) with confirmed treatment failure to first-line regimens met the inclusion criteria. Each case was then matched by lottery method with two controls of similar gender and age (± 5-year range) at treatment initiation. Ultimately 248, 192, 144, and 128 HIV-positive patients on first-line treatment with no history of treatment failure were included as controls from each hospital respectively (Fig. 1).

### Data collection tool and procedure

Data was collected via a checklist from the registry database that is routinely filled in a special form for every patient upon enrollment and follow-up. The checklist was structured in a systematic way that would enable data collectors to retrieve data in an orderly fashion and detect systematic errors. All information properly labeled by the HIV-Unit in the four hospitals as per the ICD-10 standard [International classification for treatment failure] was extracted. A team of 17 professionals including; three principal investigators, four supervisors, and ten registered nurses conducted data extraction. The training was provided for the study team before the start of data collection. Onsite routine data monitoring for completeness was conducted with a regular checklist by the principal investigators throughout the data collection period (January 2021 to May 2021). Double data entry was employed using CSPro 7 for quality control.

### Study outcome

The primary outcome of this study was to determine the factors associated with TF. Moreover, we attempted to describe the patient’s demographic characteristics (sex, age, address, educational status, marital status, employment, and care entry point), and baseline characteristics (prior exposure to ART, baseline CD4^+^ cell count, WHO clinical stage, Initial cART backbone, and baseline functional status. Variables including TB status, cART changes, cotrimoxazole prophylaxis (CPT), comorbid conditions, weight shift (enrollment weight from the current weight), low-level viremia, persistent viremia, previous hospitalizations, and their adherence were also explored as independent predictors of TF.

### Operational definition

The following definitions were used:

A. Late presentations are defined as ≤ 350 CD4^+^cells/µL and/or AIDS-defining disease in WHO stage III/IV [14].
B. A case of TF was defined as a patient who fulfills any definition of TF and/or has been switched to a second line due to TF with adherence support [15].

- Clinical TF: New or recurrent clinical event indicating advanced or severe immunodeficiency (WHO clinical stage 3 and 4 clinical conditions except for TB) after 6 months of effective treatment.
- Immunologic TF: persistent CD4^+^ levels < 200 cells/mm^3^ despite treatment for 6 months.
- Virologic TF: plasma VL > 1000 copies/ml based on two consecutive VL measurements (after 3 months) with adherence counseling between measurements.

3. Adherence was assessed at each follow-up visit as good, fair, and poor if a patient missed < 5%, ≥ 5% and < 10%, and ≥ 10% doses respectively of the expected monthly doses based on pill count.

- Suboptimal adherence comprises any patient with at least one record of fair or poor adherence.

### Data processing and analysis

All analyses were conducted using SPSS version 26 for windows (SPSS Inc., Chicago, Illinois, USA). Where appropriate, demographic and HIV-related characteristics of patients were summarized using percentages, medians [± (IQR)], or mean ± standard deviation (SD). Descriptive analyses were stratified by therapy outcome in all key variables at baseline using Pearson’s Chi-square test or Fisher’s exact test, and the Mann–Whitney U test for continuous data. Normality tests were performed before running any statistical computations. CD4^+^ cell count recovery rate was calculated using the median rate of increase from treatment initiation to 6 months, 6–12 months; 12–24 months, and 24–36 months of treatment. A nonparametric test (Mann–Whitney U test) was used to compare CD4^+^ cell counts as well as CD4^+^ recovery rates between cases and controls in the specified periods. Multivariate logistic regression (Backward: Conditional) analysis was subsequently undertaken—only variables with a P-value of ≤ 0.25 in the bivariate analysis were included in the model. The potential for collinearity was minimized by not including pairs of variables with a Spearman r > 0.60 in the same model. Results are presented as crude odds ratios (cOR) and aOR with 95% CIs. A P-value < 0.05 was considered as significant.

## Results

### Demographic and clinical characteristics of patients

Among the 1068 participants (356 cases and 712 controls), females account for 45.2% and median age at enrollment was 46 years (IQR: 39–51 years) ranging from 18 to 86 years. Males were older at enrollment than females (39 years [IQR: 33–43] vs 32 years [IQR: 27–37]), P-value < 0.001. VCT was a commoner mode of entry into HIV care for controls than cases (51.1% vs 61.5%, P < 0.001) (Table1). The median time to cART failure was 37 months (IQR: 24–47 months). At baseline, participants had a median CD4^+^ cell count of 170 cells/µl (IQR = 84.5–275) (males: 150.5 cells/µl (IQR: 68–246) vs females: 187 cells/µl (103–297) and advanced HIV (CD4^+^ cell count < 200 cells/ul) was present in 621 (58.1%). The frequency of late presentation (LP) was 919 (87.0%). In the period before 2005, 2005–2010, 2010–2014, and 2014–2020, the frequencies were 82 (91.1%), 453 (92.6%), 281 (85.4%), and 103 (69.6%), respectively. Among the LPs, 303 (28.3%) presented very late (CD4 + cell count 201–350 cells/μl or WHO clinical stage III), and 621 (58.1%) presented extremely late (CD4 + cell count ≤ 200 cells/μl and/or WHO clinical stage IV) for HIV care.

**Table 1:** Baseline and clinical characteristics of adults living with HIV in Asmara’s Follow-up hospitals, Asmara, Eritrea (2001–2020)

Characteristics	Total N (%)	Cases N (%)	Controls N (%)	Chi-square (*χ*2)	*P* value
Gender					
Male	483 (45.2)	161 (44.1)	322 (45.2)	0.0	1
Female	585 (54.7)	195 (55.9)	390 (54.8)		
Cohort age					
< 25 years	167 (15.6)	59 (16.6)	108 (15.2)	1.8	0.40
26–45 years	780 (73.0)	251 (70.5)	529 (74.2)		
> 45 years	122 (11.4)	46 (12.9)	76 (10.7)		
Address					
Maekel	931 (87.1)	308 (86.5)	623 (87.4)	0.2	0.7
Outside Maekel	138 (12.9)	48 (13.5)	90 (12.6)		
Educational level					
No formal education	59 (5.5)	21 (5.9)	38 (5.4)	0.1	0.93
Primary & junior	455 (42.6)	152 (42.7)	303 (42.7)		
Secondary & above	551 (51.5)	183 (51.4)	368 (51.9)		
Marital status					
Married	505 (47.2)	156 (43.9)	349 (57.1)	5.2	0.16
Single	302 (28.4)	107 (30.1)	195 (31.9)		
Divorced	98 (9.2)	41 (11.5)	57 (9.3)		
Widowed	107 (14.9)	51 (14.4)	107 (17.5)		
Employment					
Employed	611 (57.2)	201 (56.5)	410 (57.5)	0.10	0.79
Unemployed	458 (42.8)	155 (43.5)	303 (42.5)		
Care entry point					
VCT	620 (58.0)	182 (51.1)	438 (61.5)	**14.5**	**0.006**
In-Patient Wards	64 (6.0)	22 (6.2)	42 (5.9)		
Medical OPD	255 (23.9)	94 (26.4)	161 (22.6)		
Transfer In	66 (6.2)	32 (10)	34 (4.8)		
Others	63 (5.9)	26 (7.3)	37 (5.2)		
Prior exposure to ART					
Yes	135 (12.6)	295 (82.9)	629 (89.5)	**9.3**	**0.003**
No	934 (87.4)	61 (17.1)	74 (10.5)		
Baseline CD4^+^ cell count					
≤ 200 cells/μl	621 (58.1)	230 (65.2)	391 (55.2)	**9.6**	**0.002**
> 200cells/μl	440 (41.2)	123 (34.8)	317 (44.8)		
Initial WHO clinical stage					
Stage I	923 (87.1)	250 (70.6)	673 (95.3)	**137.8**	**< 0.001**
Stage II	59 (5.6)	44 (12.4)	15 (2.1)		
Stage III	41 (3.9)	25 (7.1)	16 (2.3)		
Stage IV	37 (3.5)	35 (9.9)	2 (0.3)		
Initial regimen backbone					
D4T + 3TC	425 (39.8)	174 (49)	251 (35.2)	**40.10**	**< 0.001**
AZT + 3TC	374 (35.0)	112 (31.6)	262 (36.7)		
TDF + FTC	239 (22.4)	51 (14.4)	188 (26.4)		
TDF + 3TC	17 (1.6)	12 (3.4)	5 (0.7)		
ABC + 3TC	13 (1.2)	6 (1.7)	7 (1)		
NNRTI					
EFV	465 (43.5)	120 (34.1)	345 (48.5)	**19.9**	**< 0.001**
NVP	598 (55.9)	232 (65.9)	366 (51.5)		
cART changes					
Yes	802 (75.0)	40 (11.3)	226 (31.7)	**52.9**	**< 0.001**
No	266 (25.0)	315 (88.7)	487 (68.3)		
Current WHO clinical stage					
Stage 1	982 (87.1)	250 (70.6)	673 (95.3)	**137.8**	**< 0.001**
Stage II	59 (5.6)	44 (12.4)	15 (2.1)		
Stage III	41 (3.9)	25 (7.1)	16 (2.3)		
Stage IV	37 (3.5)	35 (9.9)	2 (0.3)		
TB Status					
No symptom	187 (17.5)	52 (14.6)	135 (18.9)	**8.9**	**0.030**
Took IPT	783 (73.2)	261 (73.3)	522 (73.2)		
Took AntiTB	28 (2.6)	15 (4.2)	13 (1.8)		
Took IPT & ANTI-TB	71 (6.6)	28 (7.9)	43 (6)		
Cotrimoxazole prophylaxis					
Yes	522 (49.4)	217 (61.3)	305 (43.4)	**30.0**	**< 0.001**
No	534 (50.6)	137 (38.7)	397 (56.6)		
Missed hospital appointments					
Yes	603 (56.4)	247 (70.8)	356 (50.2)	**40.3**	**< 0.001**
No	455 (42.6)	102(29.2)	353 (49.8)		
Record of Suboptimal Drug adherence					
Yes	609 (57.2)	248 (71.3)	361 (51)	**51.6**	**< 0.001**
No	447 (42.3)	100 (28.7)	347 (49)		
Drug Intolerance					
cART	289 (90.6)	104 (89.7)	185 (91.1)	6.2	0.1
Cotrimoxazole	23 (7.2)	7 (6)	16 (7.9)		
Anti-TB	7 (2.2)	5 (4.3)	2 (1)		
Baseline functional status					
Working	808 (75.6)	236(66.5)	572(81)	**27.7**	**< 0.001**
Ambulatory	212 (19.8)	97(27.3)	115(16.3)		
Bed-ridden	42 (3.9)	22(6.2)	20(2.7)		
Comorbid conditions					
Yes	85 (8)	44(12.4)	41(5.8)	**14.1**	**< 0.001**
No	982 (91.9)	312(87.6)	670(94.2)		
cART changes to a similar Analogue					
Yes	283 (26.5)	100 (28)	183 (25.6)	0.72	0.4
No	786 (73.5)	256 (72)	530 (74.4)		
Weight shift					
Positive	829 (79.1)	276 (78.6)	553 (79.3)	0.07	0.8
Negative	219 (20.9)	75 (21.4)	144 (20.7)		
Previous Hospitalizations					
Yes	196 (18.6)	259 (50)	108 (15.2)	**30.22**	**< 0.001**
No	860 (81.4)	259 (50)	601 (84.8)		

Bold values are designed to attract attention of the reader i.e. easy identification of significant differences

*TDF* Tenofovir disoproxil fumarate, *FTC* Emtricitabine, *AZT* Zidovudine (AZT), *3TC* Lamivudine, *ABC* Abacavir, *D4T* Stavudine, *NVP* Nevirapine, *EFV* Efavirenz, *IPT* Isoniazid Prophylaxis therapy, *OPD* Outpatient department, *VCT* Voluntary Counseling and Testing center, *SMT TMP* Sulfamethoxazole Trimethoprim

ART, antiretroviral therapy; Cases, plasma HIV RNA > 1000 copies/mL; Control: HIV RNA ˂ 1000 copies/mL)

NNRTI, non-nucleoside reverse transcriptase inhibitor; NRTI, nucleoside reverse transcriptase inhibitor

### First-line ART treatment-related condition

Median duration on first line regimen was 44 months (IQR: 20–76 months). The most frequently used first-line cART backbones were: d4T + 3TC in 425 (39.8%) followed by; AZT-3TC, 374 (35%) and TDF + FTC, 239 (22.4%). Others were TDF + 3TC, 17 (1.6%) and ABC + 3TC, 13 (1.2%). Majority of the cases were initially placed on d4T + 3TC (49%) and most of the controls were started on AZT + 3TC (36.7%), P < 0.001. Among the controls, 124 (11.9%) had viremia (plasma HIV-RNA ≥ 50–999 copies/mL [10] at the most recent visit), and 553 (78.1%) had viral suppression (< 50 copies/mL) [10] in the most recent VL test (2019-early 2020). Among cases, the median viral load was 39,810 copies/mL (6615.50–195,199 copies/mL).

**Fig. 2:**
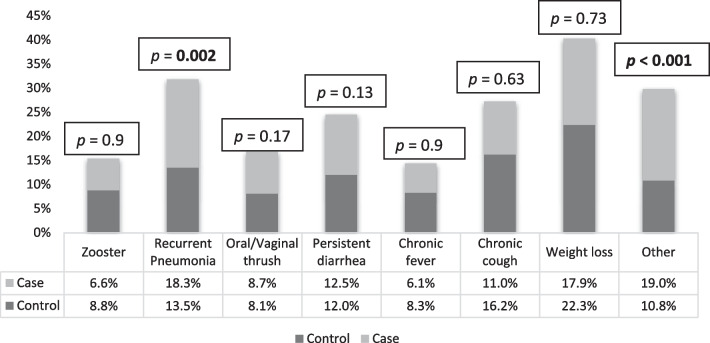
Frequency of specific comorbidities among adults living with HIV in Asmara’s Hospital Follow-up Clinics, Asmara, Eritrea (2005–2020). Others: Kapasi sarcoma, Pneumocystis pneumonia, Cytomegalovirus infection, and malignancy

Further, 75% of the study subjects had a history of cART changes with 26.5% changing to similar analogs. The cART changes were mostly prompted by adverse drug reactions/toxicity, 297 (27.8%) and drug stock-outs, 280 (25.6%). The median interval between HIV diagnosis and cART initiation was 3 months (IQR: 1–9) before 2005. Between 2005 and 2010 it was 2 months (IQR: 0.00–14), and it was 5 months (IQR: 0.00–45.5) between 2010 and 2016. As of 2016 though, the time to initiation is 1 month (IQR: 0.00–53). Regarding opportunistic infections and events among the study participants the most frequent was weight loss (n = 138), followed by recurrent pneumonia (n = 103), chronic cough (n = 95), oral/vaginal thrush (n = 56), herpes zoster (n = 53), chronic fever (n = 50) and others (n = 94). Among the study participants, 9.3% were treated with anti-TB during their follow-up while 73.2% and 50% of the participants had received Isoniazid and Cotrimoxazole prophylaxis respectively. See Fig. 2 and Table 1 for additional information.

### Multivariable analysis of predictors of first-line cART treatment failure

Multivariable logistic regression analysis was undertaken to identify the predictors of TF. In this analysis, a higher odd of TF was associated with use of specific NRTI as backbones (AZT + 3TC: aOR = 2.7 95% CI: 1.65–4.41, P-value < 0.001) (ABC + 3TC: aOR = 4.7; 95% CI: 1.18–18.82, P-value = 0.028) (aOR = 5; 95% CI: 3.03–8.2, p-value < 0.001) with TDF + FTC as reference. Other factors included prior exposure to ART (aOR = 2.28; 95% CI: 1.35–3.86, P value = 0.002); record of sub-optimal adherence (aOR = 3.08; 95% CI: 2.22–4.28, P value < 0.001); ambulatory/bedridden status at presentation (aOR = 1.61; 95% CI: 1.12–4.28, P value = 0.010); presence of comorbidities (aOR = 2.37; 95% CI: 1.36–4.10, P value = 0.002); duration on cART (< 5 years = aOR = 5.90; 95% CI: 3.95–8.73, P value < 0.001); use of SMX/TMP prophylaxis (aOR = 2.0, 95% CI, 1.44–2.78, P value < 0.001). Last but not least, baseline immune-status (CD4 + cell count) was also a predictor of TF, with those lower CD4^+^ cell having lesser odds of failure than higher CD4^+^, see Table 2. A separate multivariable logistic regression was also conducted to evaluate for sociodemographic determinants of TF. Although in univariate analysis participants who were divorced showed 1.6 times higher odds of TF compared to those married, none of the variables remained significant in multivariate analysis.

**Table 2:** Multivariable analysis of Clinical predictors of treatment failure among adults living with HIV in Asmara Hospital Follow-up Clinics, Asmara, Eritrea (2001–2020)

Variables	Case vs Controls			
	**cOR (95% CI)**	**P value**	**aOR (95% CI)**	**P value**
NRTI				
TDF + FTC	1	**< 0.001**	1	**< 0.001**
AZT + 3TC	2.41(1.40–4.16)	**0.002**	2.70(1.65–4.41)	**< 0.001**
ABC + 3TC	4.38(1.10–17.51)	**0.037**	4.73(1.18–18.92)	**0.028**
D4T + 3TC	4.07(2.2.12–7.79)	**< 0.001**	5.00(3.03–8.20)	**< 0.001**
Prior exposure to ART				
No	1	0.115	1	**0.002**
Yes	1.78(0.87–3.64)		2.28(1.35–3.86)	
Care entry point				
VCT	1	0.426		
In patient	0.749 (0.38–1.48)	0.749		
Medical OPD	1.11 (0.76–1.61)	0.380		
Others	1.61 (0.81–3.18)	0.155		
Transfer in	1.62 (0.66–3.95)	0.291		
NNRTI				
EFV	1	0.310		
NVP	1.25 (0.81–1.94)			
Record of Sub-optimal Drug adherence				
No	1	**< 0.001**	1	**< 0.001**
Yes	3.06 (2.20–4.26)		3.08 (2.22–4.28)	
Baseline functional Status				
Working	1	**0.010**	1	**0.010**
Ambulatory/Bed-ridden	1.63 (1.12–2.37)		1.61 (1.12–2.31)	
Any comorbidities				
No	1	**0.001**	1	**0.002**
Yes	2.55 (1.46–4.45)		2.37 (1.36–4.10)	
Duration of cART				
> 5 years	1	**< 0.001**	1	**< 0.001**
< 5 years	5.93 (3.95–8.90)		5.90 (3.95–8.73)	
SMX TMP				
No	1	**< 0.001**	1	**< 0.001**
Yes	2.01 (1.44–2.81)		2.00 (1.44–2.78)	
Baseline CD4 + cell count				
< 50 cells/ µl	1	**0.039**	1	**0.039**
> 50–100 cells/ µl	1.91 (1.11–3.26)	**0.019**	1.92 (1.12–3.28)	**0.017**
> 100–200 cells/ µl	1.05 (0.63–1.72)	0.974	1.07 (0.65–1.76)	0.787
> 200–350 cells/ µl	1.55 (0.91–2.20)	0.305	1.57 (0.929–2.66)	0.092
> 350 cells/ µl	1.13 (0.58–2.20)	0.992	1.12 (0.57–2.18)	0.745
The duration between diagnosis and initiation of cART	0.992 (0.986–1.00)	**0.007**	0.992 (0.986–1.00)	**0.006**

Bold values are designed to attract attention of the reader i.e. easy identification of significant differences

*TDF* Tenofovir disoproxil fumarate, *FTC* Emtricitabine, *AZT* Zidovudine (AZT), *3TC* Lamivudine, *ABC* Abacavir, *D4T* Stavudine, *NVP* Nevirapine, *EFV* Efavirenz, *OPD* Outpatient department, *VCT* Voluntary Counseling and Testing center, *SMT TMP* Sulfamethoxazole Trimethoprim, *CI* confidence interval

ART, antiretroviral therapy; Cases, plasma HIV RNA > 1000 copies/mL; Control: HIV RNA ˂ 1000 copies/mL);

NNRTI, non-nucleoside reverse transcriptase inhibitor; NRTI, nucleoside reverse transcriptase inhibitor

### Longitudinal changes in absolute CD4 + cell count

**Fig. 3:**
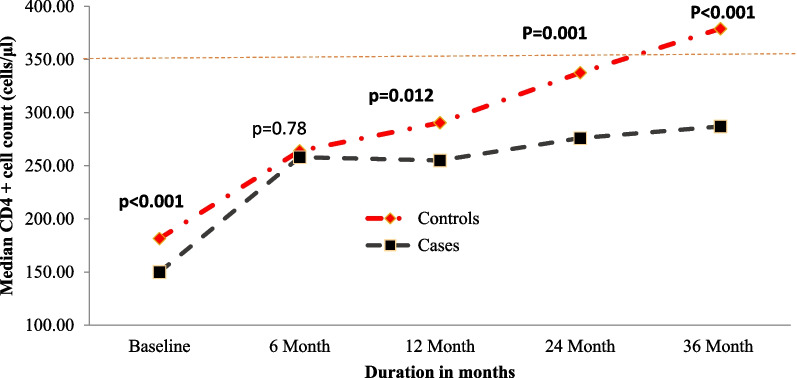
Graph showing the median CD4^+^ cell count at baseline and different periods of treatment, stratified by therapy outcome status among adults living with HIV in Asmara’s Hospital Follow-up Clinics, Asmara, Eritrea (2005–2020)

The median CD4 + cell count at baseline was significantly lower for cases than controls (150.0 cells/ µl; IQR = 70.5–259.0) vs (181.5 cells/ µl; IQR = 95.3–286.0), P < 0.001 (Mann Whitney–U test). After 6 months of treatment, both cases and controls showed similar median CD4 + cell count, cases showed a marked response to 258 cells/ µl (IQR = 148.0–367.0) almost catching up to that of controls which amounted to 264 cells/µl (IQR = 150.3–375.5), P = 0.787. However, after a year of treatment the median CD4 + cell count among cases declined to 255 cells/ µl (IQR = 148.5–369.5) while it increased to 290.5 cells/µl (IQR = 187.00–406.25) among controls (P = 0.012). Moreover; two years following treatment with cART the difference became even more pronounced, with the median count for cases only increasing to 276.0 cells/µl (IQR = 179.8–445.0). In contrast, median CD4 + cell count for controls showed marked recovery increasing to 337 cells/µl (IQR = 220.0–480.3) (P = 0.001). In the exact same manner, 3 years following treatment a similar pattern in immune response to treatment was observed, cases had a median count of 287.0 cells/µl (IQR = 186.5- 425.0) whereas controls had around 379.0 cells/µL (IQR = 259.0–515.5), with a P value < 0.001. (Fig. 3). Data on CD4 + cell count kinetics are shown in Table 3.

**Table 3:** Median CD4^+^ cell count in Cells/ µl /month (IQR) change per month during the first 3 years of treatment initiation among adults living with HIV in Asmara’s Hospital Follow-up Clinics, Asmara, Eritrea (2005–2020)

Median Rate in cells/ µl /month (IQR)	Rate 1: Baseline–6 Months	Rate 2: > 6 Months–12 Months	Rate 3: > 12 Months–24 Months	Rate 4: > 24 Months–36 months
Case	21.6 (4.4–40.2)	1.3 (− 5.8–10.5)	2.3 (− 4.6–8.3)	0.7 (− 4.5–6.4)
Control	17.6 (4.7–32.3)	5 (− 2–12.5)	4 (− 0.5–10.4)	3.1 (− 2–8.4)
*P value	0.34	**0.058**	**0.034**	**0.003**

Bold values are designed to attract attention of the reader i.e. easy identification of significant differences

*Mann Whitney–U test

## Discussion

This retrospective analysis of data from multiple HIV/AIDs treatment sites is not unique in exploring the factors associated with TF in patients from the region. What makes it unique is the focus on patients in Eritrea. Importantly, the combination of factors associated with TF was unique. In this regard, it adds a unique context to TF literature from the region. Overall, multiple determinants of TF were uncovered. Specifically, TDF + EFV-based-cART appeared to be more effective than non-TDF-based regimens (AZT + 3TC, ABC + 3TC, and D4T + 3TC). These findings align with previous studies which suggested that DRMs to TDF are limited [16] and that their safety record is relatively good [17]. Importantly, reports suggest that the efficacy of AZT, D4T, and ABC-based cART has been compromised by the emergence of DRMs in the region [16]. In addition, studies on NVP-based cART vs EFV-based ART have suggested that the former is marginally less efficacious [18]. However, our analysis did not uncover such associations.

Multiple explanations can be invoked to explain why TDF + EFV-based cART is less associated with TF. First, adverse effects (vomiting, diarrhea, among others) associated with AZT, ABC, and D4T may potentially compromise treatment or account for differences in adherence rates. Importantly, a study in Kenya demonstrated that TDF had a lower drug change rate (1.9 per 100 person-years) compared to D4T (27 per 100 person-years) [19]—the changes were largely attributed to adverse drug reactions (mostly lipodystrophy and polyneuropathy). Interestingly, adverse drug reactions were a prominent reason for changes in initial cART in this setting. Another possibility with far-reaching implications is the presence of resistance mutations (RMs). Incredibly, research suggests that ≥ 50–90% of patients experiencing virologic failure on first-line cART with viral count > 1000 copies/ml have NNRTI resistance [20, 21]. In particular, patients in SSA with first-line TF to NNRTI + NRTIs often present with the M184V mutation (associated with NRTIs − 3TC/FTC) and the K103N mutation (NNRTIs) [22, 23]. Resistance testing is not part of the treatment protocol in Eritrea as a drug resistance survey conducted in 2017 revealed RM less than 10% among newly diagnosed, which is less than the WHO threshold for routine resistance assay.

However, indirect evidence of the potential role of DRMs was noted in this study. First, large proportions of patients (77.4%) were on 3TC-based regimens and suboptimal response to treatment in patients who were switched to 3TC-based regimens was also noted, this is in line with some literature [24]. More importantly, patients were placed on failing regimens for an extended duration of time. Petersen et al. demonstrated that delayed second-line ART switch has been associated with the emergence of DRMs [25]. A different line of evidence that points to the possible presence of DRMs was the observed relationship between prior antiretroviral drug use (PAU) and TF. In general, prior exposure to ART—regardless of viral load count—has been linked to an increased likelihood of TF [22]. Others have also suggested that the increased risk of TF observed in patients with PAU is largely attributable to pre-treatment drug resistance (PDR) [26]. According to this report, HIV-infected adults in SSA starting first-line NNRTI-based cART and have a history of PAU, i.e. ART or single dose Nevirapine (sdNVP) for PMTCT, were more likely to have TF [26]. In addition, Cutrell and Jodlowski noted  that in the absence of resistance testing, it’s prudent to assume resistance to drug regimens with relatively low genetic barriers to resistance, such as EFV, 3TC, FTC, Raltegravir (RAL), Elvitegravir (EVG), if these agents were part of a previously failing regimen [10]. Therefore, the WHO recommended the use of DTG-based regimens as the preferred first-line cART regimen in a key programmatic shifts [8]. As previously noted, this recommendation has not been implemented, fully to be precise, in some settings in the region. In Eritrea, most patients were on non-DTG-based regimen as of 2020. Therefore, this study highlights the need to expedite the implementation of the WHO 2018 treatment guidelines which recommended DTG-based regimens as the preferred first-line regimen.

Similar to other studies in the region [26, 27], we noted that sub-optimal adherence to cART was a principal contributor to TF. Sub-optimal adherence can lead to high healthcare costs, poor patient outcomes (increased HIV-related morbidity and mortality), the emergence of DRMs, and increased community HIV transmission [10]. Numerous studies have demonstrated that even in high-functioning health systems, adherence to cART remains a major obstacle in HIV/AIDS treatment programs [10]. For instance, a recent study in Ethiopia suggested that the likelihood of TF was 5.4 fold higher among those who had poor adherence [29]. Likewise, poor adherence to cART as a correlate of TF was reported by investigators in Kenya [30] and Ethiopia [27, 28]. In summary, we can maintain that despite the relative heterogeneity of study designs and the diverse nature of backgrounds studied in SSA; inadequate adherence to cART is a ubiquitous contributor to TF in the region. Multiple socio-demographic, environmental, and behavioral factors are known to influence sub-optimal adherence. These include older age, living conditions/situation, stigma, early-stage HIV infection, comorbid mental health conditions, DRMs, adverse drug effects, drug-drug interaction, poor tolerability, polypharmacy drug stock-outs and substance use [10]. To understand and address the challenges associated with sub-optimal cART adherence, a better appreciation of the relevant determinants is required. Unfortunately, it can be argued that while most studies in the region highlight the importance of sub-optimal adherence to cART; its determinants are poorly described [31]. In this regard, the study corroborates the findings of a recent meta-analysis which identified toxicity as a prominent cause of poor adherence, 58% (95% CI: 46, 69%; Range: 14.4–88.5%) [32]. Beyond these issues, concerns regarding the diagnostic accuracy of self-reported adherence data have been highlighted [33]. That said, the inability to obtain a reliable quantification of the adherence process over time can thus be a barrier to intervention. These concerns are highly relevant in this setting.

By most accounts, the problem of poor adherence to cART in treatment programs in SSA is formidable. However, success has been demonstrated for mitigation efforts that prioritize the integration of adherence interventions as part of routine clinical care. In the United States, cART adherence is discussed at every visit, and patients triaged as poor adherers are promptly referred for counseling or enhanced adherence intervention or support [20]. Alternatively, some authors support the idea that chronic non-cART adherers should be placed on regimens with a higher barrier to resistance-boosted protease inhibitors (PI) or Dolutegravir (DTG) [10]. In SSA, the latter option will require faster implementation of the 2018 WHO guidelines or expansion of cART choices.

Further, we demonstrated that low CD4^+^ cell count (baseline CD4^+^ cell count of < 50 and ≤ 100 cells/ µl) were associated with increased odds of TF. Comparable results have been reported in Ethiopia [27, 34–36], and Kenya [37], among others. To explain this relationship, the inverse relationship between CD4^+^ cell count and viral replication at specific stages of the disease has been invoked [34]. In addition, a low CD4^+^ cell count is a marker of advanced disease, hence the potential presence of HIV-defining infections. In important respects, the foregoing discussion underscores the fact that delayed/late diagnosis/presentation [919 (87.0%) presented late] is one of the biggest problems facing HIV treatment programs in Asmara, Eritrea. Admittedly, problems related to study design may undermine the accuracy of the late presentation estimates. Either way, we believe that our estimates are largely reliable. Apart from CD4^+^ cell count, surrogate pointers to the late presentation as a major problem in this setting can be gleaned from several associations in the bivariate analysis. These include the proportion of patients with WHO Stage III and IV disease at baseline, baseline functional status, comorbidities, and the inverse relationship between time of HIV diagnosis and initiation of cART. Predictably, some of these factors emerged as predictors of TF in the multivariable model. Overall, we can conclude that the observed gap between HIV diagnosis and treatment for some patients requires particular scrutiny since it can compound the problem of late presentation.

Previous studies have shown that late entry to care is harmful in multiple ways—worse prognosis, shorter survival, and less benefit from cART [38, 39]. Documented factors associated with late presentation include male gender, older age, stigma, poor mental health [38, 39], low-risk perception, discrimination, lack of spousal HIV status disclosure, lower income, poor social support, level of education, lack of awareness about the need for early HIV, access to testing and treatment sites, limited investments in the community, and structural interventions [37–44]. Thus, drivers of late presentation are from diverse domains (economic, social, demographic, geographic, and psychosocial) and are undeniably complex and contextualized. As such, no two settings share the same complement of factors. This argument underscores the importance of local data. Unfortunately, the factors associated with late presentation are under-described in Eritrea. Thus, efforts to improve early HIV diagnosis (e.g. mobile- and home-based testing and counseling), early linkage to chronic HIV care centers, and timely initiation to cART should be prioritized. Formulation of new treatment models for late presenters should also be addressed (particularly CD4^+^ cell count ≤ 200 cells/μl and/or WHO clinical stage III and IV). There is strong evidence from the region indicating that intervention models mandating weekly or bi-weekly contact with care sites can work. These models are generally credited with early identification and treatment of opportunistic infections (OIs) and reductions in morbidity and mortality.

To further understand the relationship between treatment and immunological response. We considered it important to evaluate the kinetics of CD4^+^ cell count at specified intervals. In the process, we noted some outstanding points. Immunological recovery (> 500 cells/mm^3^) was generally poor in a majority of patients (cases: 14% vs Control: 28%), who achieved it after 36 months of treatment. This was contrary to the conclusions in a recent review which noted that achievement of sustained virologic suppression with cART is typically associated with a steady increase in peripheral blood CD4^+^ cell count recovery (> 500 cells/ µl) [10]. They also noted that ~ 15–20% of patients, particularly late presenters who start therapy at CD4^+^ cell count (< 200 cells/ µl) will plateau at a CD4^+^ cell count below the immunological recovery threshold. Our results are generally in line with the latter conclusion. More importantly, we also demonstrated that the rate of CD4^+^ cells/ µl /month increase differed significantly between cases and controls—a finding that is by no means unique [43]. In all, the overall CD4^+^ cells/ µl /month was substantially less than what is recommended by some investigators/or guidelines. We can infer based on these results that poor recovery of the immune system remaisn a problem in patients on cART in Asmara. Therefore, to improve outcomes and avoid or delay hard clinical endpoints, these patients should be placed under enhanced monitoring. This conclusion is supported by studies which have suggested that  CD4^+^ cell count gains < 100 cells/μl/year can be used to identify patients at risk of hard endpoints such as AIDS, serious non-AIDS events, and death [43].

Finally, we have to note that our model suggests that Cotrimoxazole prophylaxis (CPT) was a predictor of virologic failure. Relatable finding was previosly reported in a study conducted in Ethiopia [44]. According to these investigators, the relationship was potentially linked to the fact that CPT can prevent OIs  thereby leading to a  reduction in the incidence of virologic failure. An alternative, equally plausible view, relates to the fact that patients experiencing TF are more likely to present with OIs and are thus more likely to be placed on CPT by clinicians. Therefore, the finding merely raises questions regarding triggers for CPT. 

### Strengths and limitations of the study

To the best of our knowledge, our study is the first to evaluate the factors associated with virologic failure in Eritrea. Regardless, it has several limitations. First, the study uses secondary data collected retrospectively. This approach has been associated with the incompleteness of clinical data. Moreover, underreporting/missing data elements can lead to biases—particularly if they are systemic. Secondly, the contribution of HIV drug resistance to TF was not assessed. Lastly, adherence information was largely based on self-report. However, recall and social desirability bias may undermine the reliability of this approach. Despite these limitations, we would like to highlight some strengths: first, information on a large number of variables including demographic information; care entry point; prior exposure to cART; date of HIV diagnosis; date of cART initiation and subsequent treatment history; and clinical outcomes were collected. The availability of this information permitted an in-depth analysis of multiple secondary objectives. Second, the sample size was fairly large for multiple variables thereby strengthening the robustness of our results.

## Conclusion

This multi-center analysis demonstrates that the HIV/AIDS treatment program in Asmara, Eritrea requires optimization in multiple domains. Unlike other countries in the region, patients in this setting are older. At baseline, the majority of the participants presented with advanced HIV, thereby making late presentation a major problem with important individual and public health consequences. Further, it can be asserted that although viral suppression was achieved in a significant number of cases, immunological recovery was poor. Multivariate analysis demonstrated that multiple modifiable risk factors were associated with an increased likelihood of TF. Looking into the future, we believe that there is a need for additional resources and efforts targeted at the optimization of cART adherence, diversification of cART regimens, and interventions directed at enhancing early HIV diagnosis and prompt initiations of treatment. Robust evaluation and monitoring in the first years following treatment initiation will be paramount to detecting poor treatment response and subsequent action, including surveillance of DRMs are needed.

## Data Availability

The dataset supporting the conclusions of this article is available from the corresponding author on reasonable request.
